# At It Again

**Published:** 1902-02

**Authors:** 


### AT IT AGAIN.

THE rivalry in the veterinary profession to exceed all others in the production of an extensive literature on rabies and hydrophobia has broken out again. This time in Pennsylvania. We have not heard recently from Massachusetts on this subject. The Bay State for many years was the storm centre of this agitation, but it has moved south and west, and the District of Columbia and Pennsylvania threaten to rob the effete East of its laurels in this direction. We hope it won't reach Chicago, where it would be sure to assume tornado-like characteristics. The Western plains are just beyond.

